# Paediatric gastric organoids as a tool for disease modelling and clinical translation

**DOI:** 10.1007/s00383-020-04821-x

**Published:** 2021-01-25

**Authors:** Brendan C. Jones, Giuseppe Calà, Paolo De Coppi, Giovanni Giuseppe Giobbe

**Affiliations:** 1 Stem Cell and Regenerative Medicine Section, Developmental Biology and Cancer Research and Teaching Department, Great Ormond Street Institute of Child Health, University College London, London, UK; 2 Department of Specialist Neonatal and Paediatric Surgery, Great Ormond Street Hospital, London, UK

**Keywords:** Gastric, Development, Organoids, Regenerative medicine

## Abstract

**Purpose:**

Knowledge of gastric epithelial homeostasis remains incomplete, lacking human-specific models for study. This study establishes a protocol for deriving gastric epithelial organoids from paediatric gastric biopsies, providing a platform for modelling disease and developing translational therapies.

**Methods:**

Full-thickness surgical samples and endoscopic mucosal biopsies were obtained from six patients. Gastric glands were isolated by a chemical chelation protocol and then plated in 3D culture in Matrigel^®^ droplets in chemically defined medium. After formation, organoids were passaged by single cell dissociation or manual disaggregation. Cell composition and epithelial polarity of organoids were assessed by bright field microscopy and immunofluorescence analysis, comparing them to native paediatric gastric tissue.

**Results:**

Gastric glands were successfully isolated from all six patients who were aged 4 months to 16 years. Gastric glands from all patients sealed to form spherical gastric organoids. These organoids could be passaged by manual disaggregation or single cell dissociation, remaining proliferative up to 1 year in culture. Organoids retained normal epithelial cell polarity, with the apical surface orientated towards the central lumen. Organoids expressed markers of mature gastric epithelial cell types, except for parietal cells.

**Conclusion:**

Gastric organoids can be reliably generated from paediatric biopsies and are a representative in vitro model for studying gastric epithelium.

## Introduction

The microscopic morphology of gastric glands in humans has been well described with surface (pit), neck, and basal domains [1]. The pit domain is composed mainly of mucus secreting cells, while the neck and base contain other mucin producing cells, enteroendocrine, chief, and, in the body of the stomach, parietal cells. However, while much is known about intestinal development and epithelial homeostasis in humans [2, 3], our knowledge of these processes in the gastric epithelium remains incomplete. Historically, studies of stomach development have relied on animal models, usually in rodents [4–6]. Stem and progenitor cell populations have been identified in mice [4, 5] and transcription factors controlling regionalisation during murine gut tube development have been defined [6]. However, rodent stomach contains a forestomach lined by squamous epithelium, a different structure from humans, forcing caution in generalising these data to human development and disease [1]. Morphological studies of human embryos, diseased gastric tissue, and gastric epithelial cell lines derived from malignant tissue provide some human-specific insights but have significant ethical limitations or fail to represent normal homeostasis, respectively. A high-fidelity, reproducible in vitro system is therefore preferable for understanding human development and homeostasis.

McCracken and colleagues have derived human gastric organoids by directed differentiation of induced pluripotent stem cells (iPSC) [7, 8]. However, the protocol relies on timed exposure of the iPSC to patterning signals based on studies of murine gastric development and by inhibition of known pro-intestine forming signals, our knowledge of which remains incomplete [7]. While these iPSC-derived gastric organoids contain both epithelium and stroma and will prove useful for studying mechanisms of development, once formed they are not readily useful for therapeutic purposes.

After discovery of the endodermal epithelial stem cell marker *Lgr5* by the Clevers group [2], epithelial organoids have been derived and characterised for most endodermal abdominal organs, including adult gastric organoids [9, 10]. The behaviour of these adult gastric organoids mirrors other *Lgr5*-stem cell-based organoid systems in that they are easily expandable, genetically stable in culture, and can be used to model disease [11]. To this point, these systems have used adult tissue derived from partial or total gastrectomy and have focussed on the study of adult disease.

Herein we present a reliable methodology for obtaining human gastric epithelial organoids from paediatric stomach samples, including 3–5 mm endoscopic mucosal biopsies. These organoids represent an adaptation of the epithelial organoid system to study gastric epithelial homeostasis and disease in children. We show that this system is feasible using small starting tissue volumes, which can be obtained with minimal morbidity to the child. Therefore, gastric organoids derived in this manner offer the potential to model paediatric mucosal diseases and subsequently could form a platform for cell-based regenerative medicine therapies.

## Materials and methods

### Ethics statement

Endoscopic mucosal and full-thickness surgical paediatric gastric biopsies were collected after an informed consent process with an independent research coordinator, in compliance with license 18DS02.

### Isolation of paediatric gastric glands from surgical and endoscopic specimens

**Fig. 1:**
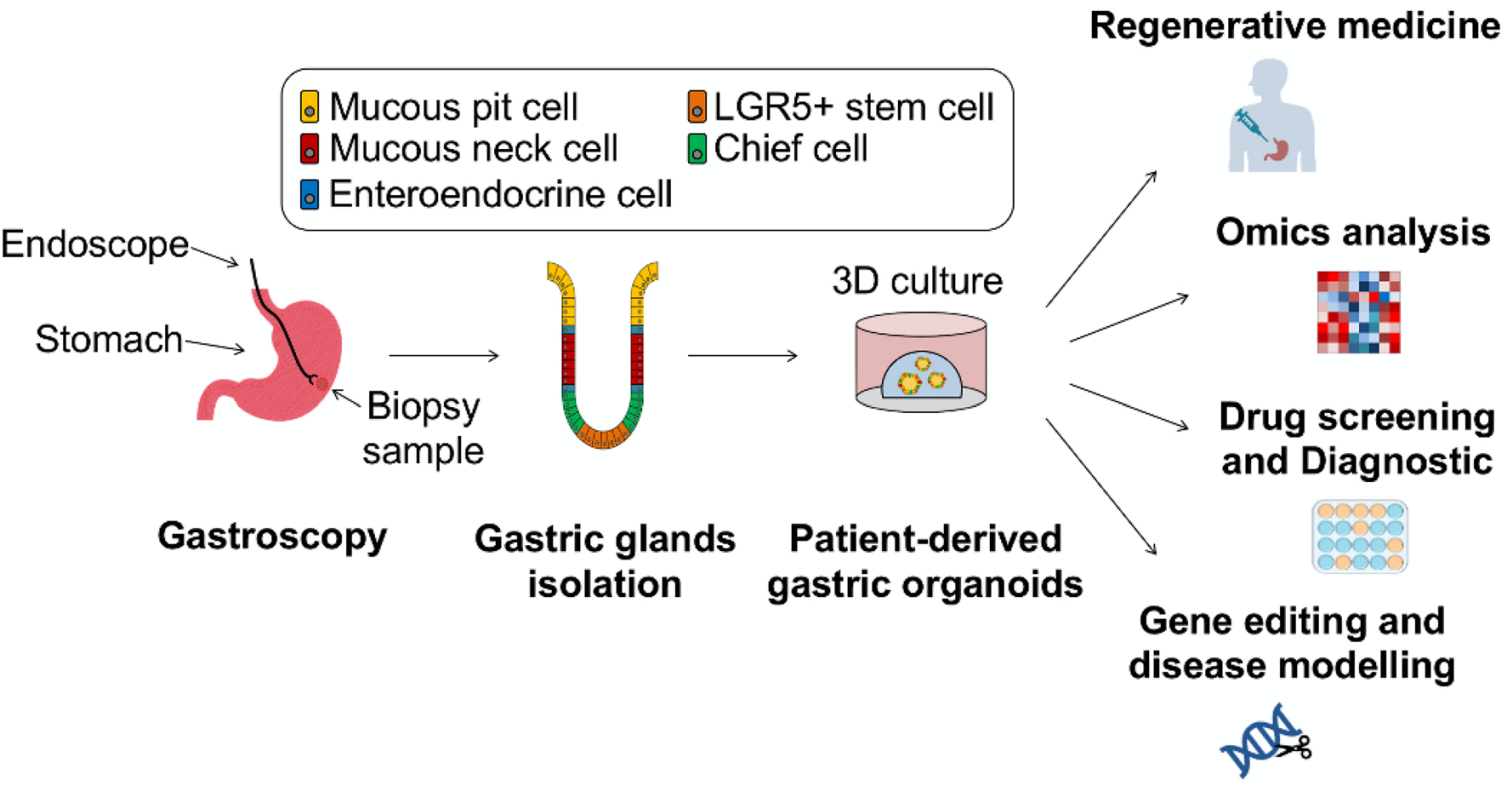
Schematic depicting the workflow for isolation of human paediatric gastric organoids from endoscopic biopsies and the potential applications of these patient-derived organoids

Figure 1 shows an overview of the gastric gland isolation and organoid culture process, as well as the potential applications of paediatric gastric organoids. Human paediatric gastric tissues, either endoscopic mucosal biopsies or full-thickness surgical specimens, were collected in ice-cold sterile Advanced DMEM F-12 (ADMEM/F12) basal medium (Thermo Fisher, 12634). Tissue was transported to laboratory on ice and processed within 1 hour of collection. Gastric gland stem cells were isolated from specimens using an adaptation of published dissociation protocols for adult tissues [9, 11].

Tissue was washed in cold Hank’s balanced salt solution (HBSS; Thermo Fisher, 88284) in a petri dish. The luminal mucus layer was then removed by scraping the mucosa with a glass coverslip. For full thickness specimens, the mucosa and muscle layers were separated by blunt dissection. Isolated mucosa was then cut into pieces < 5 mm in diameter. Mucosal pieces were washed vigorously with HBSS using a 10 mL pipette pre-coated with 1% bovine serum albumin (BSA; Sigma-Aldrich, A9418), discarding the supernatant after allowing the pieces to settle. This process was repeated until the supernatant was clear.

Mucosal pieces were then incubated in a chelating buffer containing 5.6 mmol/L Na_2_HPO_4_, 8.0 mmol/L KH_2_PO_4_, 96.2 mmol/L NaCl, 1.6 mmol/L KCl, 43.4 mmol/L sucrose, 54.9 mmol/L D-sorbitol, 0.5 mmol/L DL-dithiothreitol, and 2 mM EDTA (all from Sigma-Aldrich) dissolved in MilliQ water (18.2 mΩ/cm) for 30 min at 37 °C on a shaking platform. Pieces were transferred to a dry sterile petri dish on ice and glands were released from the mucosa using direct pressure with a glass coverslip. Glands were collected by washing the petri dish with ice cold ADMEM/F12 plus 1% penicillin–streptomycin (Thermo Fisher, 15140122), 10 mM HEPES (Thermo Fisher, 15630080), and 2 mM Glutamax (Thermo Fisher, 35050061) (called “ADEM/F12+++”). The gland-containing medium was passed through a 40 μm cell strainer and centrifuged at 300*g* for 5 min at 4 °C. After aspirating the supernatant, the cell pellet was resuspended in liquid growth factor reduced Matrigel® basement membrane matrix (Corning, 354230). Droplets of 30 μL were plated in warmed multi-well tissue culture plates. The plates were then inverted and incubated for 15 min at 37 °C to induce gelation of the Matrigel®, with gravity allowing the glands to be drawn towards the medium-facing surface of the inverted Matrigel® droplet during gelation. After gelation, a chemically defined, Good Manufacturing Practice (GMP)-compliant, human gastric organoid medium was added to the well (see below), along with Rho kinase inhibitor (Y-27632; Tocris, 1254) if single cells were visible in the droplet. Glands were cultured in normoxia with 5% CO_2_ and medium was changed every 3–4 days. For small endoscopic biopsies, plating in a single 30 μL droplet was appropriate, while full thickness surgical specimens, such as gastrostomy closures, yielded enough glands to plate three or more 30 μL droplets of appropriate density.

Human paediatric gastric organoid medium includes “ADMEM/F12+++” (as above), 1X B-27 supplement without vitamin A (Thermo Fisher, 12587010), 1.25 mM N-acetylcysteine (Sigma Aldrich, A9165), 100 ng/mL Wnt-3A (Peprotech, 315–20), 500 ng/mL R-spondin 1 (Peprotech 120–38), 100 ng/mL Noggin (R&D Systems, 6057-NG), 50 ng/mL epidermal growth factor (EGF) (Thermo Fisher, PMG8043), 10 nM gastrin (Sigma Aldrich, G9020), 3 μM glycogen synthase kinase 3 (GSK-3) inhibitor (CHIR99021) (Tocris, 4423), 5 μM transforming growth factor beta (TGFβ) inhibitor (A83-01) (Sigma Aldrich, SML0788), and 200 ng/mL fibroblast growth factor 10 (FGF10) (Peprotech, 100–26) [12].

### Passage of organoids

After formation of organoids using the protocol described above, organoids were passaged in culture every 6–8 days by one of two methods: (1) manual disaggregation in a narrowed glass pipette, (2) enzymatic dissociation to single cells.

#### Manual disaggregation

Manual disaggregation was used as the standard method for organoid passaging during culture. Matrigel® droplets and medium were retrieved from the well by scraping and aspiration with a pipette and transferred to sterile tubes on ice. Cells were washed with 10 mL of cold ADMEM/F12+++ and centrifuged at 200*g* at 4 °C for 5 min. The pellet of organoids was resuspended in 2 mL of ice-cold ADMEM/F12+++ and then manually disrupted by repeated pipetting using a narrow-tipped glass pipette pre-coated in 1% BSA in phosphate buffered saline (PBS; Sigma-Aldrich, D8537), which applied a shear stress to fragment the organoids. Pre-coating the glass pipette was essential to avoid adhesion of organoids to the glass. Medium was topped up to 10 mL, disaggregated organoids were centrifuged again, and supernatant was aspirated until the cell pellet was near-dry.

#### Single cell dissociation

Dissociation to single cells allowed for rapid expansion in organoid number, as each dissociated progenitor cell has the potential to form a new organoid [10]. Organoids were collected from the plate and washed as above. The cell pellet was then resuspended in 1 mL of TrypLE Express (Thermo Fisher, 12605010) and incubated for 5 min at 37 °C. After incubation, organoids were pipetted again and 10 mL of ice-cold ADMEM/F12+++ was added to inactivate the TrypLE Express. The cells were then centrifuged and the supernatant aspirated.

Near-dry pellets of single cells or disaggregated organoids were then resuspended in Matrigel® at the desired split ratio (usually 1:3–1:6), plated in 30 μL droplets, inverted, and allowed to gelate, as above. Gastric organoid medium was added after 15 min and changed every 3–4 days. Rho kinase inhibitor was added to the medium of single cells immediately after plating, but not during subsequent medium changes.

### Immunofluorescence staining

Human gastric tissues were fixed in 4% paraformaldehyde (PFA; Sigma-Aldrich, 100496) for 30 min to 2 h, depending on the size of the sample, and then washed extensively in PBS. Samples were dehydrated overnight in 30% sucrose and then embedded in PolyFreeze tissue freezing medium (Polysciences, 25113) over dry ice. Sections were cut at 7 μm on a Bright Instruments cryostat and stored at − 20 °C until staining.

Organoids at 7 days from last passage were removed from Matrigel® by incubation in Cell Recovery Solution (Corning, 354253) for 45 min at 4 °C. This treatment released the organoids from the Matrigel® without damaging the structure of the organoids allowing whole mount immunofluorescence staining. Organoids were collected, washed in PBS, and transferred to 1% BSA pre-coated 1.5 mL Eppendorf tubes, and resuspended in 4% PFA for 20 min in rotation. PFA was removed and quenched with 0.1 M NH_4_Cl (Sigma-Aldrich, 254134) for 1 h in rotation to decrease aldehyde-related autofluorescence. Quenched organoids were stored in PBS with 1% penicillin–streptomycin until staining.

Native gastric mucosal sections were blocked and permeabilised with 0.5% Triton X-100 (Sigma-Aldrich, 100496) with 1% BSA in PBS for 2 h at room temperature. Organoid whole mounts were treated the same way but were maintained in rotation during this time. Primary antibodies were diluted in blocking buffer and incubated with sections or organoids for 24 h at 4 °C (static for sections, in rotation for organoids). Samples were then extensively washed in 0.5% Triton X-100 in PBS. Secondary antibodies were diluted and applied to samples as for primary antibodies and incubated overnight at 4 °C. Organoids in suspension were transferred to a glass-bottomed Petri dish immediately prior to imaging.

Primary antibodies used in this study were chromogranin A (Abcam, ab15160), mucin 5AC (Invitrogen, ma5-12178), mucin 6 (Abcam, ab216017), pepsinogen C (Abcam, ab9013-1), gastrin (Leica, PA0681), all at 1 in 100 dilution, except gastrin and pepsinogen C which were used at 1 in 500. Secondary antibodies were AlexaFluor® anti-rabbit 488 (Thermo Fisher, A11008), AlexaFluor® anti-rabbit 568 (Thermo Fisher, A11011), AlexaFluor® anti-mouse 488 (Thermo Fisher, A11001), and AlexaFluor® anti-sheep 488 (Thermo Fisher, A11015) all at 1 in 200 dilution. AlexaFluor® Phalloidin 647 (Thermo Fisher, A22287) was used at 1 in 100 dilution and Hoechst 33342 (Thermo Fisher, H1399) at 10 μg/mL.

### Image acquisition

Freshly isolated gastric glands and organoids were imaged during cell culture in bright field using a Zeiss Axio Observer A1. Immunofluorescence images of native gastric mucosal sections and organoid whole mounts were acquired on a Zeiss LSM710 confocal microscope.

## Results

### Gastric organoids can be successfully derived from paediatric gastric biopsies

**Fig. 2:**
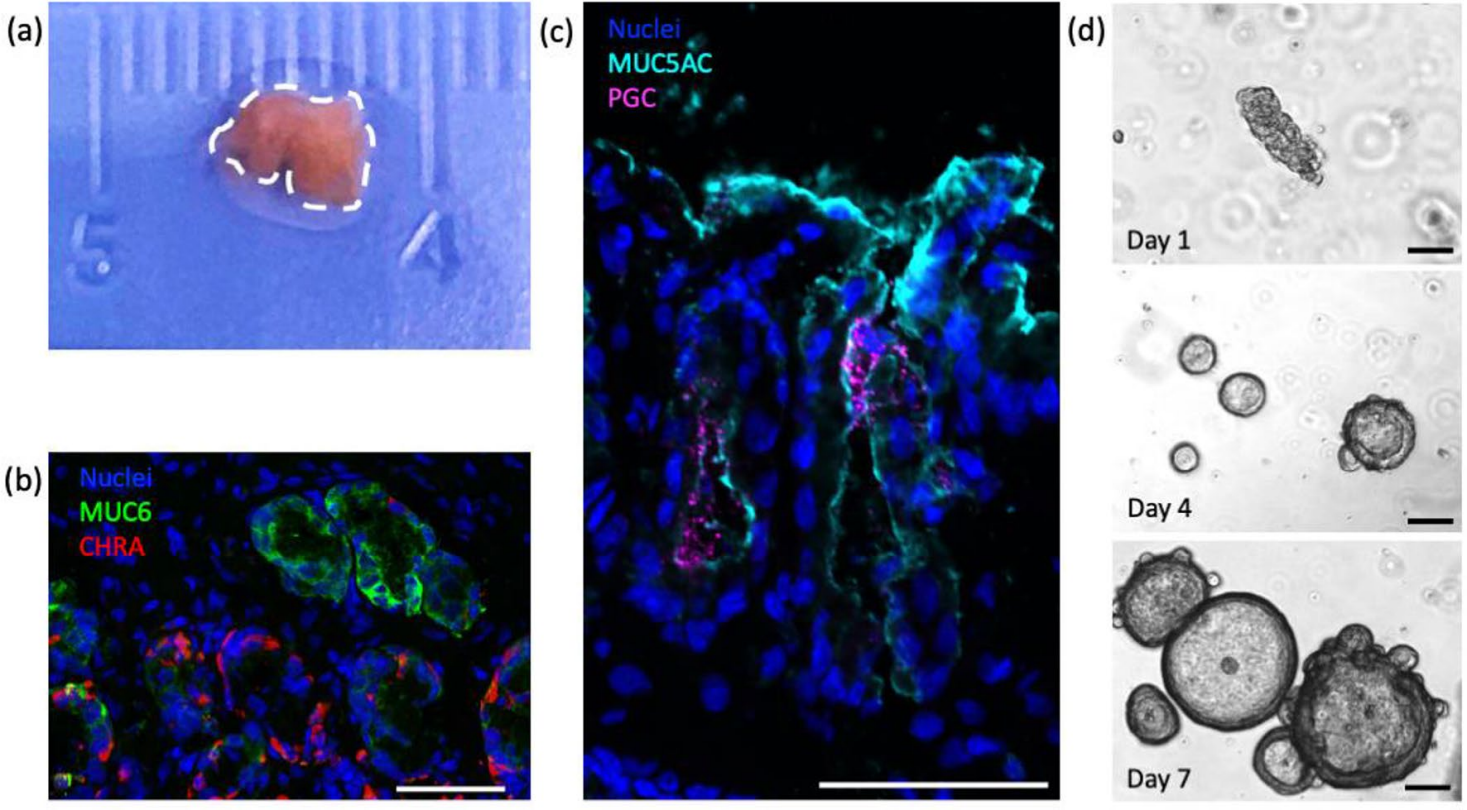
**a** Endoscopic biopsy of gastric mucosa from a paediatric patient prior to processing. **b**, **c** Characterisation by immunofluorescence of gastric mucosa from endoscopic biopsies. Mucin 6 (MUC6) in green, chromogranin A (CHRA) in red, Mucin 5AC (MUC5AC) in cyan, pepsinogen C (PGC) in magenta, and nuclei in blue (Hoechst). Scale bars 50 μm. **d** Bright field images of gastric glands isolated from endoscopic biopsies forming epithelial gastric organoids when cultured in chemically defined medium (detailed in methods). Scale bars 100 μm

Gastric tissue samples were obtained from six patients ranging in age from 4 months to 16 years. No patients had primary diseases of the stomach at final diagnosis. Two of the samples were endoscopic mucosal biopsies (Fig. 2a) and the other four were full-thickness surgical specimens. As expected, paediatric gastric mucosa contained mature surface (pit) mucin producing cells (mucin 5AC, MUC5AC), gland (neck) mucin producing cells (mucin 6, MUC6), enteroendocrine cells (chromogranin A, CHGA), and chief cells (pepsinogen C, PGC) (Fig. 2b, c).

Gastric glands were successfully isolated from all six patient samples and encapsulated in Matrigel® (Fig. 2d, top panel). By day 2–4 in culture, elongated gastric glands sealed and formed cystic structures with a central cavity lined by a single layer of cells (Fig. 2d, middle panel). Organoids formed from each of the six patients. These spherical structures closely resemble the morphology of adult epithelial gastric organoids [9]. The organoids continued to proliferate and enlarge over the subsequent days, with some evidence of gland-like budding after day 7 in culture (Fig. 2d, bottom panel). Non-gland cells encapsulated in the Matrigel® died during the first 7 days in culture. These cells were likely either non-epithelial or non-progenitor epithelial cells, and as such were not supported by the gastric organoid-specific medium.

### Paediatric gastric organoids can be maintained in culture and rapidly expanded

**Fig. 3:**
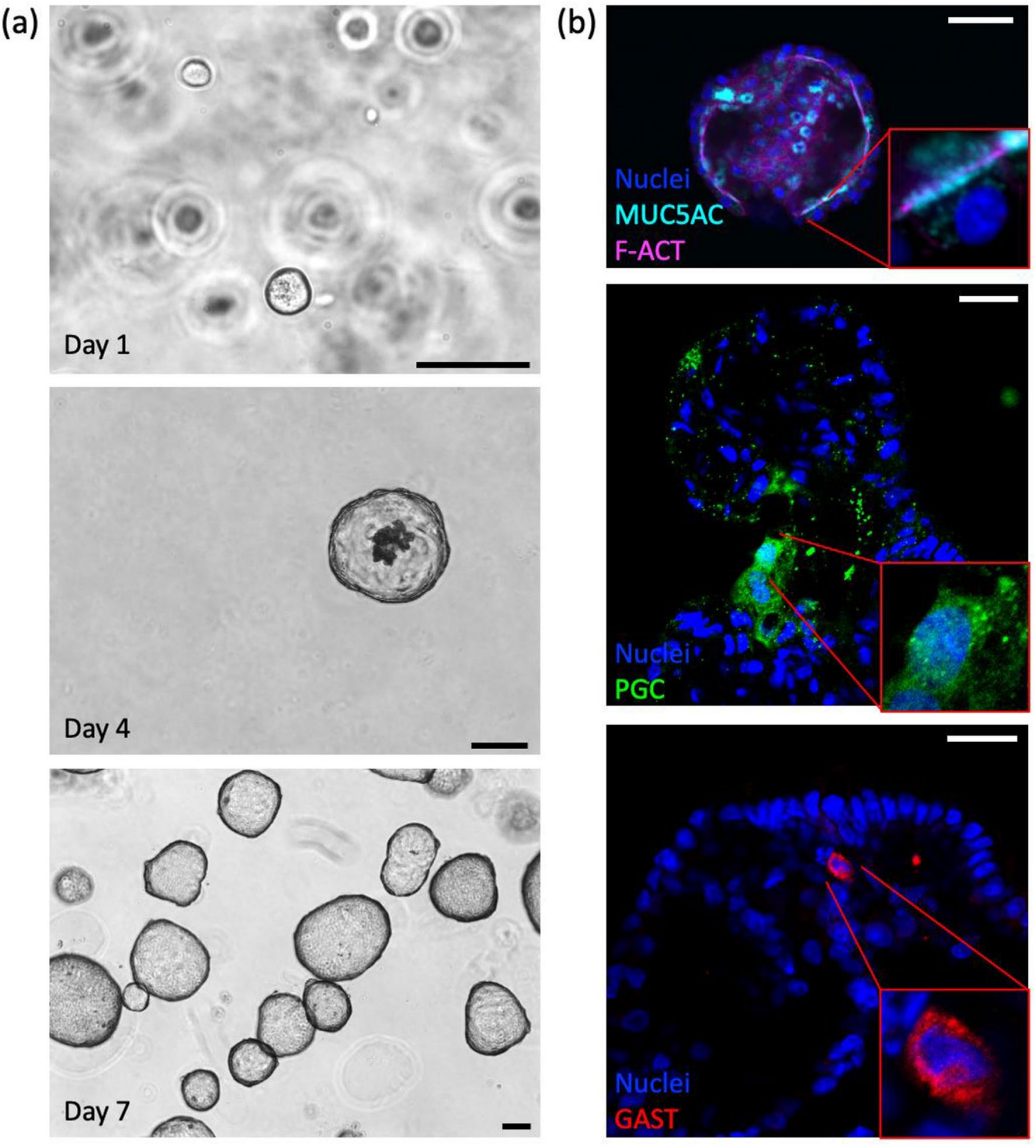
**a** Bright field images of paediatric gastric organoids in culture. Single cells (top panel) form organoids within 7 days of seeding in Matrigel® (middle & bottom panels). Scale bars 100 μm. **b** Immunofluorescence panel showing gastric organoids with appropriate polarity and expression of mature cell types. Mucin 5AC (MUC5AC) in cyan, f-actin (F-ACT) in magenta, pepsinogen C (PGC) in green, gastrin (GAST) in red, and nuclei in blue (Hoechst). Scale bars 50 μm

Following derivation from gastric glands, gastric organoids could be passaged using manual disaggregation or by dissociation to single cells. Figure 3a shows a typical passage following single cell dissociation and culture with rho kinase inhibitor, which was crucial to the formation of organoids from single cells (data not shown). By day 4 from dissociation to single cells, spherical organoids reliably formed with a central lumen. As the culture progressed, sloughed cells appeared in the lumen of the organoid consistent with epithelial turnover (Fig. 3a, middle panel). At day 7, organoids were well formed and had continued to grow in size compared to day 4, while remaining healthy and organised in appearance (Fig. 3a, bottom panel). Morphology of the gastric organoids is similar to that observed in freshly isolated gastric glands and remains unchanged up to and including passage 45 (52 weeks in culture), when the experiments were electively stopped. Single cells derived from the organoids in one 30 μL Matrigel® droplet were sufficient to form new organoids in six 30 μL droplets (1:6 split ratio) at similar density to the original droplet, demonstrating the expansion potential of this organoid culture system.

Organoids passaged using the manual disaggregation technique also re-formed organoids reliably after passaging. However, instead of being able to replate at a 1:6 split ratio as for single cell dissociation, manually disaggregated organoids were usually plated at a 1:3–1:5 ratio to preserve optimal density. Passaging in this manner avoided the need to use the anti-apoptotic rho kinase inhibitor that is required in single cell passaging.

### Gastric organoids retain cellular polarity and mature cell subtypes

Having demonstrated that gastric organoids have a morphology consistent with an epithelium-lined structure with a central lumen, we next examined cell polarity and the cellular subsets retained in the organoids. Paediatric gastric organoids showed appropriate epithelial polarity with apical surface of cells orientated towards a central lumen, as shown by localisation of f-actin to the luminal surface of the cells (Fig. 3b, top panel). The spherical morphology of the organoids suggested a substantial surface (or pit) domain and indeed there was prominent expression of the surface mucin 5AC on the luminal surface of the cells, consistent with secretion of the mucin into the central lumen of the organoids (Fig. 3b, top panel). Chief cells, marked by pepsinogen C (PGC), and gastrin-secreting enteroendocrine cells were present in the gastric organoids (Fig. 3b, middle and bottom panels). Pepsinogen C localised to the cytoplasm of chief cells in a speckled pattern, consistent with the secretory granules known to be present in chief cells in native tissue (Fig. 3b, bottom panel). Similarly, gastrin was appropriately localised to the cytoplasm of G cells (Fig. 3b, bottom panel). Chief and G cells were shown to be less common in the organoids compared to surface mucin cells, consistent with proportions observed in native stomach tissue. Parietal cells, marked by proton pump subunit ATP4B, were not observed in the organoids. However, neck mucus cells (MUC6), stem cells (Lgr5), and other enteroendocrine cell types (for example, somatostatin) were also present in the gastric organoids (data not shown). This suggests gastric organoids to be a representative in vitro model of paediatric gastric epithelium.

## Discussion and conclusions

In this study, we provide a reproducible method for the derivation of gastric organoids from infants and children (4 months to 16 years of age). Organoids can be successfully obtained from limited surgical specimens, such as gastrostomy closures, as well as from endoscopic mucosal biopsies. Organoids recapitulate the gastric epithelium of origin, including the expression of mature cell subtypes and normal apico-basolateral epithelial cell polarity. Organoids remain healthy and can be passaged in culture for up to a year without needing to obtain further tissue from the original patient. These findings represent a successful adaptation of the adult organoid system to paediatric patients [9–11].

As shown in Fig. 1, the potential applications of this system are many. The generation of organoids from patients ranging in age from infancy to post-puberty offers the potential to study homeostatic mechanisms in paediatric gastric epithelium across the developmental spectrum of childhood. This can be done through transcriptomic and metabolomic profiling, techniques that have been used by our group to study the interaction between the extracellular matrix and various endodermal organoids [12]. Little is known about any changes in gastric epithelial physiology over the early years of life and combining this system with advanced omics analysis techniques offers exciting potential to gain new insights.

Reliable derivation of organoids, even from sub-centimetre biopsies, offers the chance to obtain patient-specific organoids from children with diseases affecting the gastric mucosa and model these diseases in vitro*,* at minimal risk or morbidity to the child. Organoids from healthy adult patients have been used to investigate host–pathogen interactions, such as *Helicobacter pylori* infection [9, 11]. In light of the current global SARS-CoV-2 pandemic and the known abdominal manifestations of COVID-19 disease in children [13–15], gastric and other abdominal organoid models represent tools that could be useful in understanding the pathogenesis of emerging infections and screening potential therapies [16].

In addition to host–pathogen interactions, gastric organoids have the potential to model inherited mucosal diseases, such as hereditary diffuse gastric cancer, most commonly caused by a germline mutation in the *CDH1* gene [17]. Studying organoids derived from such patients may provide mechanistic insights into such diseases and it has been demonstrated that a similar organoid model can be applied to adult gastric cancers [9].

Moreover, from a translational and regenerative perspective, gastric organoids may prove useful as cell-based therapies. For example, it has been shown that intestinal organoids can improve mucosal healing in models of inflammatory intestinal diseases [18, 19] and be used to generate functional region-specific engineered small intestine mucosa [20]. While proton pump inhibitors represent effective treatment for gastritis in most patients, there may be a role for organoid-based therapies in refractory and severe cases, and in the setting of profound immunocompromise, such as severe mucositis following bone marrow transplantation. As gene editing technology such as CRISPR-Cas9 continues to develop, it is reasonable to hypothesise that monogenic diseases could be treated via genetic modification of patient-specific organoids [21]. Following expansion to clinically relevant numbers, autologous organoids could then be transplanted back into the patient, reducing the risk posed by the inherited disease, whilst also avoiding the complications of immunosuppressive therapy.

An alternative in vitro system to the current study involves the generation of gastric organoids from induced pluripotent stem cells (iPSC) [7, 8]. These organoids contain both epithelial and mesenchymal compartments, compared to the epithelium-only organoids of the current study. Induced PSC-derived gastric organoids are a useful tool to study molecular mechanisms underlying gastric development, as the technique was developed by using already elucidated normal mammalian gut tube morphogenic patterning signals [7]. However, the protocol is long (approximately 1 month to generate iPSC-derived gastric organoids vs less than 1 week in this study), costly, and relies on reprogramming methods to faithfully recapitulate pluripotency without affecting subsequent mature cellular phenotypes. The generation of gastric organoids from human foetal tissue could allow the study of gastric development directly from source tissue, without induced pluripotency and long differentiation protocols.

In a subsequent work [8], the same authors were able to produce iPSC-derived gastric organoids containing parietal cells, the major cell type lacking in epithelium-only organoids, including those in this study [9–11]. This was accomplished by small molecule and cytokine manipulation during the final stages of the differentiation protocol. However, once these organoids were passaged, the parietal cell phenotype was lost and could not be retrieved, evidence for the difficulty of forming a stable population of parietal cells in vitro.

In conclusion, we have demonstrated that gastric organoids can be reliably derived from paediatric gastric epithelium, including from sub-centimetre endoscopic biopsies. These organoids retain expression of tissue-specific mature cell markers and normal epithelial polarity. They can be kept in culture for extended periods and can be expanded exponentially in number. Gastric organoids represent a model to study paediatric gastric homeostasis, paediatric mucosal diseases, and ultimately develop translational therapies.

## References

[1] Kim T-H, Shivdasani RA (2016). Stomach development, stem cells and disease. Development.

[2] Barker N (2007). Identification of stem cells in small intestine and colon by marker gene Lgr5. Nature.

[3] McCracken KW (2011). Generating human intestinal tissue from pluripotent stem cells in vitro. Nat Protoc.

[4] Arnold K (2011). Sox2(+) adult stem and progenitor cells are important for tissue regeneration and survival of mice. Cell Stem Cell.

[5] Qiao XT (2007). Prospective identification of a multilineage progenitor in murine stomach epithelium. Gastroenterology.

[6] Verzi MP (2009). Role of the homeodomain transcription factor Bapx1 in mouse distal stomach development. Gastroenterology.

[7] McCracken KW (2014). Modelling human development and disease in pluripotent stem-cell-derived gastric organoids. Nature.

[8] McCracken KW (2017). Wnt/beta-catenin promotes gastric fundus specification in mice and humans. Nature.

[9] Bartfeld S (2015). In vitro expansion of human gastric epithelial stem cells and their responses to bacterial infection. Gastroenterology.

[10] Barker N (2010). Lgr5(+ve) stem cells drive self-renewal in the stomach and build long-lived gastric units in vitro. Cell Stem Cell.

[11] Schlaermann P (2016). A novel human gastric primary cell culture system for modelling *Helicobacter pylori* infection in vitro. Gut.

[12] Giobbe GG (2019). Extracellular matrix hydrogel derived from decellularized tissues enables endodermal organoid culture. Nat Commun.

[13] Xiao F (2020). Evidence for gastrointestinal infection of SARS-CoV-2. Gastroenterology.

[14] Xu Y (2020). Characteristics of pediatric SARS-CoV-2 infection and potential evidence for persistent fecal viral shedding. Nat Med.

[15] Tullie L (2020). Gastrointestinal features in children with COVID-19: an observation of varied presentation in eight children. Lancet Child Adolescent Health.

[16] Lamers MM (2020). SARS-CoV-2 productively infects human gut enterocytes. Science.

[17] Vogelaar IP (2012). Familial gastric cancer: detection of a hereditary cause helps to understand its etiology. Hereditary Cancer Clin Pract.

[18] Fukuda M (2014). Small intestinal stem cell identity is maintained with functional Paneth cells in heterotopically grafted epithelium onto the colon. Genes Dev.

[19] Yui S (2012). Functional engraftment of colon epithelium expanded in vitro from a single adult Lgr5(+) stem cell. Nat Med.

[20] Meran L, Massie I, Campinoti S, Weston AE, Gaifulina R, Tullie L, Faull P, Orford M, Kucharska A, Baulies A, Novellasdemunt L, Angelis N, Hirst E, König J, Tedeschi AM, Pellegata AF, Eli S, Snijders AP, Collinson L, Thapar N, Thomas GMH, Eaton S, Bonfanti P, De Coppi P, Li VSW (2020). Engineering transplantable jejunal mucosal grafts using patient-derived organoids from children with intestinal failure. Nat Med.

[21] Fujii M, Clevers H, Sato T (2019). Modeling human digestive diseases with CRISPR-Cas9-modified organoids. Gastroenterology.

